# Innovative cost-effective method to repair lacrimal cannaliculi laceration – finding proximal end and stent

**DOI:** 10.3205/oc000109

**Published:** 2019-05-31

**Authors:** Shreya M. Shah, Mehul A. Shah, Kashyap B. Patel, Romi U. Singh

**Affiliations:** 1 Drashti Netralaya, Dahod, Gujarat, India

**Keywords:** lacrimal canalicular laceration, prolene suture, stent, identification of proximal end

## Abstract

**Purpose:** To explore a new technique to find out the proximal end of lacerated canaliculi and a new material for the stent.

**Methods:** Surgery was performed on 9 eyes of 9 patients using a 5/0 prolene suture needle as a modified probe. Prolene suture was inserted as a stent and left in place for two months. All the data were analyzed.

**Results:** The surgery was successful in all cases and the prolene were removed after two months. The mean follow-up time after the tube removal was 3.8 months (range 3–6 months). No other complications associated with the prolene sutures were noticed except for epiphora and corneal irritation in three cases. All the tubes were removed successfully without any difficulty. No iatrogenic injuries occurred during prolene removal.

**Conclusions:** The reported surgical technique is a very cost-effective option for lacrimal canalicular laceration repair.

## Introduction

Lid lacerations are common with ocular injuries [1], [2], [3] while an association with lacrimal passage injuries is less common [1]. Canalicular injuries are relatively common, but controversy persists regarding repair and the surgical methods to be employed. In this review, various techniques are analyzed in a historical context. Recent studies of lacrimal drainage in systems with monocanalicular obstruction are cited. Various surgical techniques in the repair of canalicular injuries are reviewed, including methods of identifying the medial lacerated lacrimal passages and the various lacrimal stents that have been used. Various methods of stent placement and fixation are systematically categorized. Case series of canalicular repair reported in the literature are reviewed and the results and complications are compared [2].

## Methods

Our study was approved by the Hospital Ethical Committee. We developed a surgical technique to manage lid laceration and enrolled patients for this program.

The eyelid was repaired surgically in layers. The lacrimal canal was repaired using a very novel technique. A new method to identify the proximal and distal ends of lacerated canaliculi was identified along with a newer material of stent.

The choice of general or local anesthesia will depend on the age of the patient, the location and extent of the injury, the type of stent, and the surgeon’s experience [4]. General anesthesia is mandatory for virtually all children and for many adults. The techniques of “local” anesthesia include topical intranasal anesthetics and vasoconstrictors, a regional block of the intratracheal and anterior ethmoidal nerves as well as local subcutaneous infiltration. 

**Figure 1:**
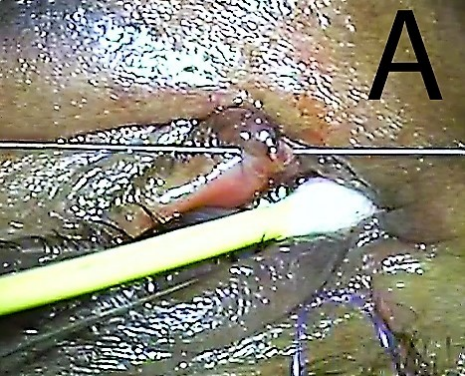
Lacrimal probe passed through lower punctum to distal end

**Figure 2:**
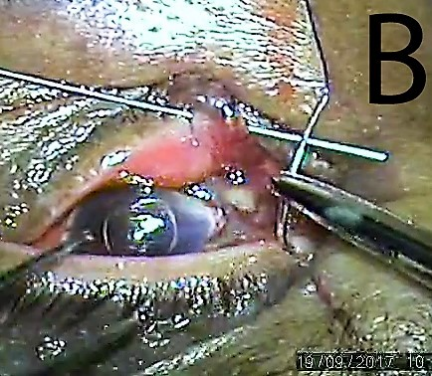
Blunt prolene needle passed through upper punctum

We penetrated the Bowmen lacrimal probe from the lower punctum (Figure 1 (Fig. 1)) and identified the distal end of the laceration (Figure 2 (Fig. 2)).

**Figure 3:**
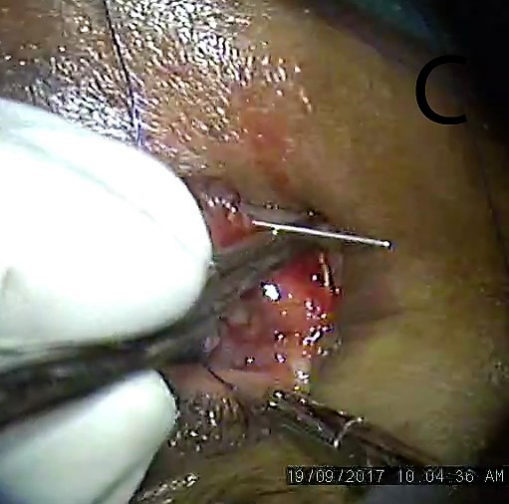
Blunt end of the needle brought out of the proximal lacerated end

We utilized the 5/0 prolene suture with spatula needle to identify the proximal end of laceration and broke the sharp tip of the needle (Figure 3 (Fig. 3)).

**Figure 4:**
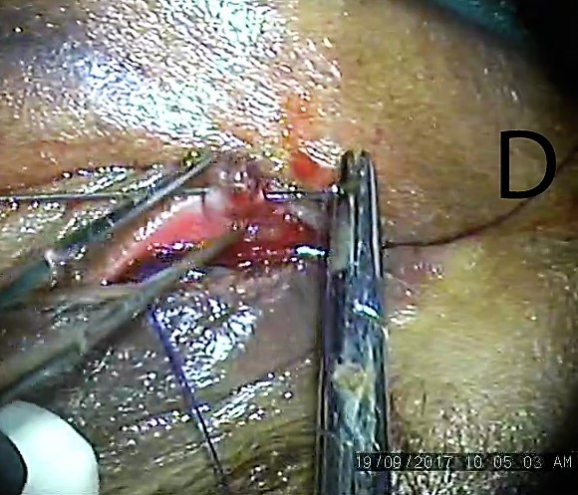
Blunt prolene needle passed through distal lacerated end using rail road technique

**Figure 5:**
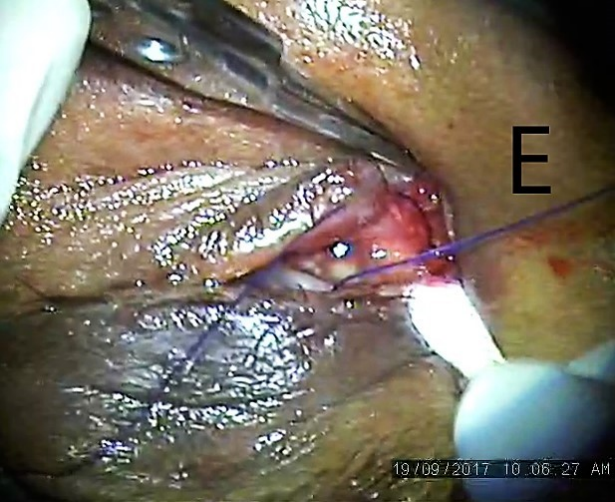
Prolene suture passed and loop created

**Figure 6:**
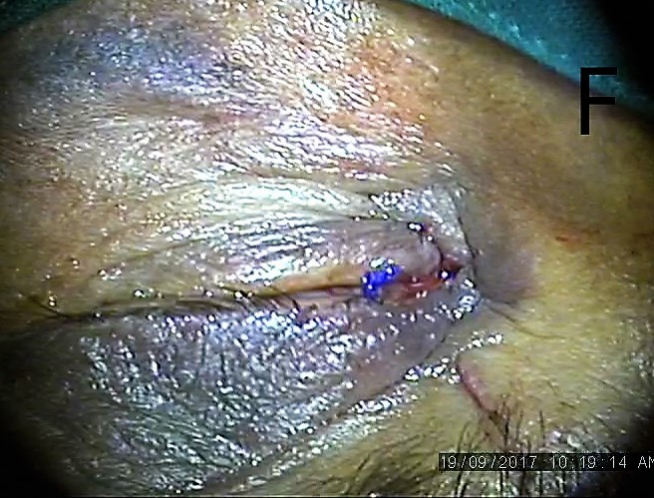
Prolene suture left in place as stent

The blunt end of the needle was inserted in the upper punctum, which was intact, was passed through the canaliculi and brought out of the proximal torn end (Figure 4 (Fig. 4)). The blunt end was followed up in the distal end with railroad technique with lacrimal probe (Figure 5 (Fig. 5)). The prolene suture was left in place as a stent for six weeks and knot was slipped in canaliculi (Figure 6 (Fig. 6)) and the rest of the lid was sutured in layers.

We removed the prolene suture after eight weeks. 

## Results

We enrolled nine cases with a mean follow-up of 98 days. The causes of canalicular laceration include fighting, dog bites, fall or collisions while running, and sticks or cattle horns. Laceration of the inferior canaliculus is more common than that of the superior canaliculus. We found all patients to be doing well anatomically in relation to integrity. We found lacrimal passages in all cases and the patients were symptom-free. 

## Discussion

Although canalicular injuries are relatively common, controversy persists regarding repair and the surgical methods to be employed. Methods of treating canalicular laceration include a laissez-faire approach, exteriorization, or anastomosis with the placement of a canalicular stent. Canalicular stents may be placed with monocanalicular, bicanalicular-annular or -nasal fixation techniques. Although a wide variety of lacrimal stents have been used in the past, most reported case series of canalicular laceration have emphasized one or more of these techniques with the use of silicone tubing as the stent material. Although iatrogenic injury of the uninvolved canaliculus and premature loss of a stent have been cited, high rates of success have been reported using these techniques and complications have been infrequent or minor [1].

Many authors have suggested the use of pig tail to detect the proximal end [4], [5], [6], [7], [8]. A similar mechanism is used in the current technique but in a more cost-effective way.

Naturally occurring organic and metal stents have generally been used in a monocanalicular fashion. The relative inflexibility of metal limits its application as a simple monocanalicular stent. More flexible, synthetic stents of nylon, polyethylene, and silicone make have been placed in either a mono- or bicanalicular fashion. Although metal canalicular stents are still in use, silicone tubing has become more popular and is viewed as the canalicular stent material of choice. This is particularly true while considering medial lacerations or tissue loss involving the common canaliculus or lacrimal sac [1]. Bicanalicular silicon tube insertion is reported to be the most accepted material and method [9], [10], [11], [12], [13], [14], [15]. Mini monoka is a monocanalicular silicone stent reported to have a high success rate and ease of insertion [16], [17], [18]. Teflon is also reported as an option but is unlikely to be used in the long-term [19].

Studies suggest a stent removal time interval of two to six months [1]; we removed the prolene suture after two months.

## Conclusions

The reported method is a unique technique to repair, which is very cost-effective, easy to adopt, and assures excellent results without much discomfort to the patient. The current procedure is an alternative solution, if a silicone tube is not possible or not available.

## Notes

### Competing interests

The authors declare that they have no competing interests.
